# Hypoxia-Induced Adaptations of N-Glycomes and Proteomes in Breast Cancer Cells and Their Secreted Extracellular Vesicles

**DOI:** 10.3390/ijms251810216

**Published:** 2024-09-23

**Authors:** Bojia Peng, Kai Bartkowiak, Feizhi Song, Paula Nissen, Hartmut Schlüter, Bente Siebels

**Affiliations:** 1 Section Mass Spectrometry and Proteomics, University Medical Center Hamburg-Eppendorf, 20246 Hamburg, Germany; pengbojia.uke@gmail.com (B.P.); p.nissen@uke.de (P.N.); b.siebels@uke.de (B.S.); 2 Department of Tumor Biology, University Medical Centre Hamburg-Eppendorf, 20246 Hamburg, Germany; k.bartkowiak@uke.de; 3 Institute of Neuropathology, University Medical Center Hamburg-Eppendorf, 20246 Hamburg, Germany; songfeizhi@gmail.com

**Keywords:** N-glycosylation, hypoxia, extracellular vesicles, N-glycomics, proteomics

## Abstract

The hypoxic tumor microenvironment significantly impacts cellular behavior and intercellular communication, with extracellular vesicles (EVs) playing a crucial role in promoting angiogenesis, metastasis, and host immunosuppression, and presumed cancer progression and metastasis are closely associated with the aberrant surface N-glycan expression in EVs. We hypothesize that hypoxic tumors synthesize specific hypoxia-induced N-glycans in response to or as a consequence of hypoxia. This study utilized nano-LC–MS/MS to integrate quantitative proteomic and N-glycomic analyses of both cells and EVs derived from the MDA-MB-231 breast cancer cell line cultured under normoxic and hypoxic conditions. Whole N-glycome and proteome profiling revealed that hypoxia has an impact on the asparagine N-linked glycosylation patterns and on the glycolysis/gluconeogenesis proteins in cells in terms of altered N-glycosylation for their adaptation to low-oxygen conditions. Distinct N-glycan types, high-mannose glycans like Man3 and Man9, were highly abundant in the hypoxic cells. On the other hand, alterations in the sialylation and fucosylation patterns were observed in the hypoxic cells. Furthermore, hypoxia-induced EVs exhibit a signature consisting of mono-antennary structures and specific N-glycans (H4N3F1S2, H3N3F1S0, and H7N4F3S2; H8N4F1S0 and H8N6F1S2), which are significantly associated with poor prognoses for breast tumors, presumably altering the interactions within the tumor microenvironment to promote tumorigenesis and metastasis. Our findings provide an overview of the N-glycan profiles, particularly under hypoxic conditions, and offer insights into the potential biomarkers for tracking tumor microenvironment dynamics and for developing precision medicine approaches in oncology.

## 1. Introduction

The growth of primary or metastatic tumors often leads to hypoxic conditions in their central regions, triggering cellular adaptation mechanisms [1]. In triple-negative breast cancer (TNBC), these adaptive responses result in a more aggressive phenotype and increased resistance to chemotherapy and radiotherapy [2]. Hence, understanding the tumor cell responses to hypoxia is crucial for developing more effective therapies to counter tumor progression and treatment resistance.

Extracellular vesicles (EVs) are nano-sized particles secreted by all cell types containing bioactive molecules such as proteins, DNA, and mRNAs, which play a crucial role in intercellular communication [3]. Tumor-derived EVs, influenced by their microenvironmental conditions, have potential as therapeutic targets or biomarkers for specific cancer types and stages [3]. Hypoxia-induced EVs carry cargo that can enhance pro-angiogenic signaling, promote epithelial-to-mesenchymal transition, facilitate metastasis, and may induce invasive behavior in breast cancer cells [4].

Glycans, carbohydrate structures that modify proteins and lipids, are formed through a tightly controlled biosynthetic pathway involving glycosyltransferases and sugar transporters [5]. These structures are predominantly found on the surface of cell membranes (glycocalyx) and are essential for cell–cell communication and interactions with the extracellular matrix [6]. In cancer, significant changes occur in different types of glycosylation, notably N-glycans, which attach to asparagine residues within the sequon motif (Asn-X-Ser/Thr) and can be categorized into truncation, high-mannose, complex/hybrid, and other structural features [7]. The surface proteins of EVs are highly glycosylated [8,9], and alterations in the N-glycan structures associated with tumor cells have been detected in cancer-derived EVs, indicating their potential utility as significant biomarkers for clinical applications [6]. EVs expressing low levels of bisecting GlcNAc-modified N-glycan significantly contributed to the carcinogenesis and metastasis of the recipient cells, underlining the crucial impact of specific N-glycan structures on cancer progression [10]. Hypoxia significantly alters the glycosylation profiles of breast cancer cells, leading to increased epithelial-to-mesenchymal transition and migration [11]. However, the implications of N-glycosylation regarding the function of hypoxia-induced EVs remain unclear and necessitate further research.

The objective of this study is to investigate the impact of hypoxia on the N-glycan profiles from the secreted EVs of breast cancer cells in vitro and determine the molecular alterations underlying these changed N-glycosylation profiles. We lowered the oxygen concentration applied to the breast cancer cells to 1% (hypoxia) and isolated the EVs of an MDA-MB-231 cell line; the control cells were kept under atmospheric air (normoxia). Using liquid chromatography–tandem mass spectrometry (LC–MS/MS), we conducted quantitative differential proteomic and N-glycomic analyses to profile the EVs and their corresponding whole cell lysates. This comprehensive approach aims to elucidate the relationship between hypoxic cancer cells and N-glycan modifications, thereby identifying the potential molecular markers and therapeutic targets associated with breast cancer progression.

## 2. Results

### 2.1. EV Isolation and Characterization

**Figure 1:**
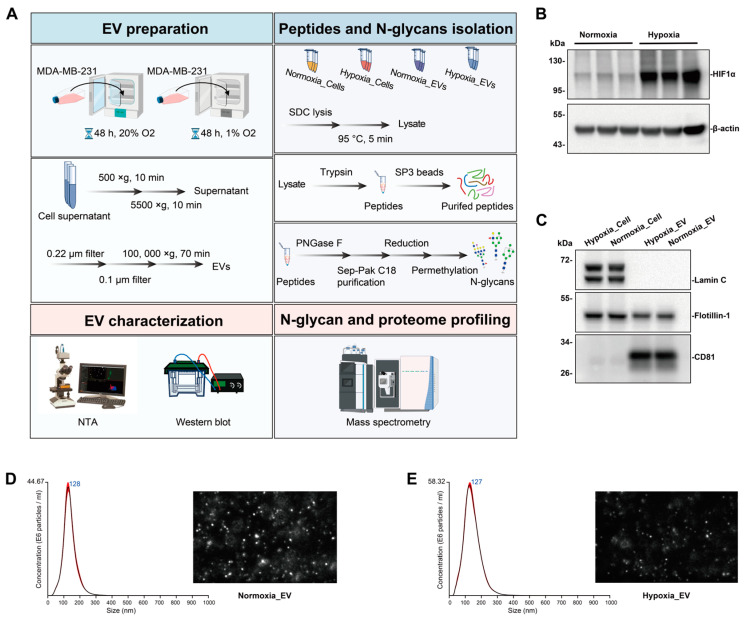
EV isolation and characterization. (**A**) Workflow for preparing EVs and conducting mass spectrometry for proteomic and N-glycomic analyses of EVs and cells. Key steps include enrichment of EVs secreted under normoxic or hypoxic conditions, isolation of peptides and N-glycans, EV characterization, and mass spectrometry analysis. (**B**) Western blot of hypoxic marker HIF1α in normoxic and hypoxic cell lysate. (**C**) Western blot of EV markers CD81, Flotillin-1, and Lamin C in normoxic and hypoxic EV lysate and cell lysate. (**D**,**E**) Size and concentration of EVs secreted under normoxia or hypoxia for MDA-MB-231 cell line, as measured by NTA. Histogram showing the calculated mean ± SD of size distribution by NTA analysis of normoxic and hypoxic EV. Red error bars indicate ± 1 standard error of the mean. Screenshots from video recorded using NanoSight LM14, showing the distribution of EVs from the cellulose-supernatant culture under normoxia or hypoxia. Three biological replicates were used for each condition.

To investigate the molecular messengers in a hypoxic breast cancer microenvironment, we analyzed the proteomes and N-glycomics of whole cell lysates and EVs from cells cultured under normoxia or hypoxia (Figure 1A). The EVs from MDA-MB-231, grown under normoxic or hypoxic conditions, were isolated by a process involving differential centrifugation followed by ultracentrifugation. The expression of hypoxia marker HIF-1α was verified in the hypoxic cell lysates (Figure 1B). The purity of the isolated EVs, generally referred to as EVs, was confirmed by the presence of the CD81 protein, which was found to be present in the MDA-MB-231-derived EVs, while Flotillin 1 was depleted in the EVs compared to its higher expression in the cell lysates, and Lamin C was undetectable in the EVs (Figure 1C). The quality and size of the EV preparations were further analyzed using NTA (Figure 1D,E), which showed a similar size distribution, with both the normoxic EVs and hypoxic EVs exhibiting a main single peak at approximately 127 nm, consistent with the typical size distribution profile of EVs [12].

### 2.2. Diversity of N-Glycome in Breast Cancer Cells and Their EVs under Normoxia and Hypoxia

**Figure 2:**
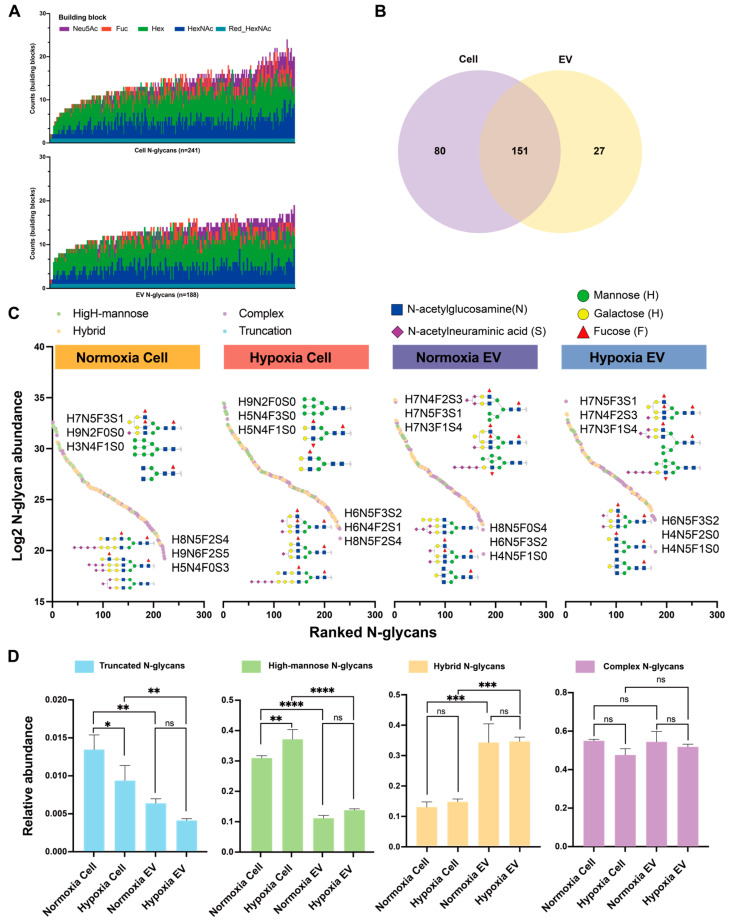
Overview of the N-glycome profiles of EVs and cells under normoxic and hypoxic conditions of the MDA-MB-231 cell line. (**A**) The stacked bar chart of identified N-glycan compositions in cells (n = 6) and EVs (n = 6). The counts indicate the number of each monosaccharide building block present in the N-glycan structures. (**B**) A Venn diagram illustrating the number of N-glycans shared between cells and EVs. (**C**) All quantified N-glycans are plotted, depicting their log-transformed relative abundances against their rank within the dynamic range. (**D**) Distribution of common N-glycosylation features in cells and EVs under normoxic and hypoxic conditions. Significant values are marked with ns (no significant), * (*p* ≤ 0.05), ** (*p* ≤ 0.01), *** (*p* ≤ 0.001), or **** (*p* ≤ 0.0001).

The N-glycomes of the cells and EVs were analyzed with a nano-LC–ESI-MS/MS system and a total of 231 and 178 N-glycan compositions were found in the cells and EVs, respectively (Table S1). To obtain a comprehensive overview of the N-glycan profiles, each N-glycan composition was plotted against its frequency of detection (Figure 2A). The Venn diagrams indicated 151 conserved N-glycans shared between the cells and EVs, with 27 N-glycan patterns unique to the EVs and 80 unique to the cells (Figure 2B). The dynamic range of the N-glycan abundance in the cells and EVs under normoxia and hypoxia spanned approximately 15 orders of magnitude (Figure 2C). N-glycans H7N5F3S1 and H6N5F3S2 were under the top highest and lowest N-glycans in abundance in at least three groups, respectively. To characterize the differences and similarities of the N-glycans in the cells and EVs under normoxia and hypoxia, quantitative differential comparisons of the identified N-glycans were performed by classifying the glycans into four glycosylation traits: truncation, high-mannose, hybrid, and complex (Figure 2D, Table S2). Truncation and high mannose were the most abundant classes in the normoxic and hypoxic cells compared to the EVs. Specifically, the truncation glycans increased in the hypoxic cells relative to the normoxic cells, while the high-mannose glycans decreased under the hypoxic conditions, but the relative abundance of the high-mannose glycans decreased in the hypoxic cells. Interestingly, the hybrid N-glycans showed the highest abundance in both the normoxic and hypoxic EVs. The pattern of the complex N-glycans showed high similarity between the EVs and cells under both the normoxic and hypoxic conditions. The N-glycans were further classified into 11 glycosylation-derived features: sialylation, fucosylation, galactosylation, number of antennas (mono-antennary, biantennary, tri-antennary, and tetra-antennary), poly-LacNAc motifs, presence of bisecting GlcNAc, and Lewis-type antigens (Figure S1). The relative abundance values of sialylation, fucosylation, galactosylation, bisection, mono-antennary, tri-antennary, and sLewis A/X were increased in the normoxic and hypoxic EVs in comparison to the normoxic and hypoxic cells, whereas the relative abundance values of poly-LacNAc and Lewis A/X were decreased in the normoxic and hypoxic EVs in comparison to the normoxic and hypoxic cells.

### 2.3. Quantitative Changes in N-Glycome in Breast Cancer Cells and Their EVs under Normoxia and Hypoxia

**Figure 3:**
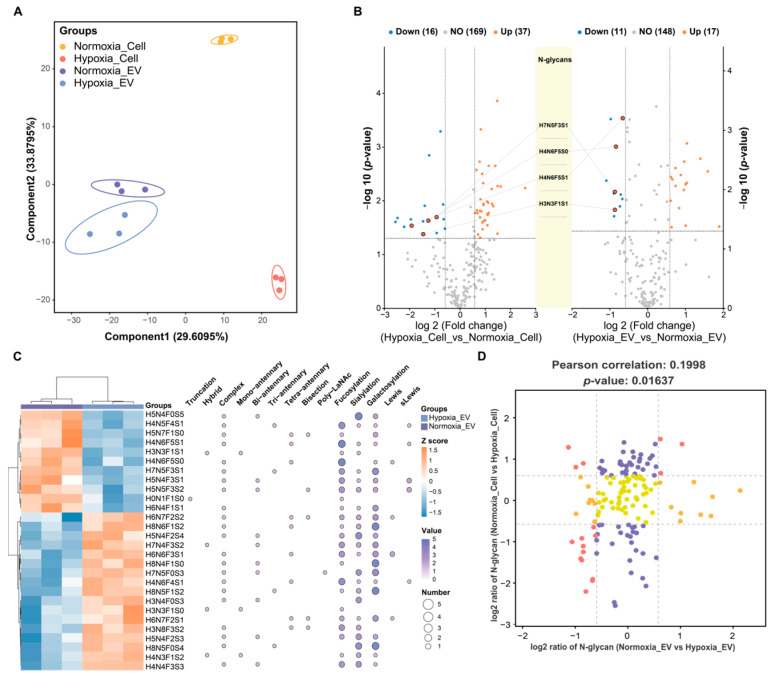
Comparison of N-glycans found in cells and EVs under normoxia and hypoxia. (**A**) Scatter plot of PLS-DA analysis of the N-glycome profile of normoxia_Cell (yellow circles), hypoxia_Cell (orange circles), and normoxia_EV and hypoxia_EV (purple and cyan squares). Symbols and ellipses indicate each sample and the 95% confidence interval of the four clusters. R^2^Y = 0.953; Q^2^Y = 0.836; *p* = 0.05. (**B**) Volcano plots showing differentially expressed N-glycans between normoxic and hypoxic conditions in both cells and EVs (*p* < 0.05 and fold change > 1.5; *q*-value < 0.05). The left panel presents a volcano plot of differentially expressed N-glycans between normoxic and hypoxic cells, while the right panel shows the differentially expressed N-glycans between normoxic and hypoxic EVs. The central diagram highlights the overlapping differentially expressed N-glycans identified in both conditions. (**C**) The heatmap for the hierarchical clustering of all *t*-test-significant N-glycans with different glycosylation traits in hypoxic vs. normoxic cells (*p* < 0.05 and fold change > 1.5; *q*-value < 0.05). Twenty-seven altered N-glycans were classified by biosynthetic class. Each N-glycan is assigned to a Ballon plot depicting glycosylation features in the respective N-glycan. (**D**) The nine-quadrant map analysis of N-glycan expression changes regarding hypoxic vs. normoxic cells and hypoxic vs. normoxic EVs. Each dot corresponds to an N-glycan. N-glycans demonstrating a significant increase or decrease in abundance (fold change > 1.5) both upon hypoxic vs. normoxic cells and hypoxic vs. normoxic EVs are shaded red. N-glycans demonstrating a significant increase or decrease in abundance (fold change > 1.5) only upon hypoxic vs. normoxic cells or hypoxic vs. normoxic EVs are shaded orange and purple. N-glycans demonstrating no significant changes regarding either hypoxic vs. normoxic cells or hypoxic vs. normoxic EVs are shaded yellow.

To investigate the differential impact of normoxic and hypoxic conditions on the N-glycomic profiles of cells and their secreted EVs, a multivariate analysis was conducted using partial least squares discriminant analysis (PLS-DA) (Figure 3A). The PLS-DA showed a clear separation of the cells and EVs under hypoxic and normoxic conditions, with component 1 accounting for 29.61% and component 2 explaining 33.88% of the variance (Figure 3A). The univariate analysis using Student’s *t*-test identified 28 altered N-glycan species between the normoxic and hypoxic EVs (*p* < 0.05; fold change > 1.5; *q*-value < 0.05) (Figure 3B; Table S3). Among them, 17 N-glycans were more abundant, and 11 were less abundant in the hypoxic EVs compared to the normoxic EVs. Interestingly, four differentially abundant N-glycans were shared between the dysregulated N-glycans in the cells under normoxic and hypoxic conditions and abnormal N-glycans in the EVs under normoxic and hypoxic conditions (Figure 3B). These N-glycans were less abundant in the hypoxic cells and EVs compared to their normoxic counterparts and contained fucose residues. Specifically, N-glycan H4N6F5S1 was modified to N-glycan H4N6F5S0 through the addition of a sialic acid residue. These statistically differentially expressed N-glycans were further analyzed using Pearson’s correlation and glycosylation features. Most of the differentially abundant N-glycans in the EVs consist of complex glycan species with fucose residues [0–5], sialic acid [0–5], galactose [0–5], bisecting GlcNAc [0–1], Lewis [0–2], and sLewis [0–2] (Figure 3C). In addition, a truncated N-glycan containing fucose resides and a glucose was lower in abundance in the hypoxic EVs, and a bi-antenna complex glycan with a poly-LacNAc epitope was lower in abundance. Interestingly, four hybrid N-glycans containing unique structural moieties were found to be higher or lower in abundance in hypoxia_EV. Furthermore, the cellular dysregulated N-glycans contained several specific structural glycosylation features (Figure S2). By distinguishing between the cellular and vesicular N-glycan expression patterns under hypoxia, the association analysis of the N-glycome data revealed a Pearson’s correlation coefficient of 0.1998 (*p*-value = 0.01637) (Figure 3D). These results explain that the abundance of most cellular differentially abundant N-glycans did not correlate with the corresponding vesicular N-glycans.

### 2.4. Proteome Profiling of EVs

**Figure 4:**
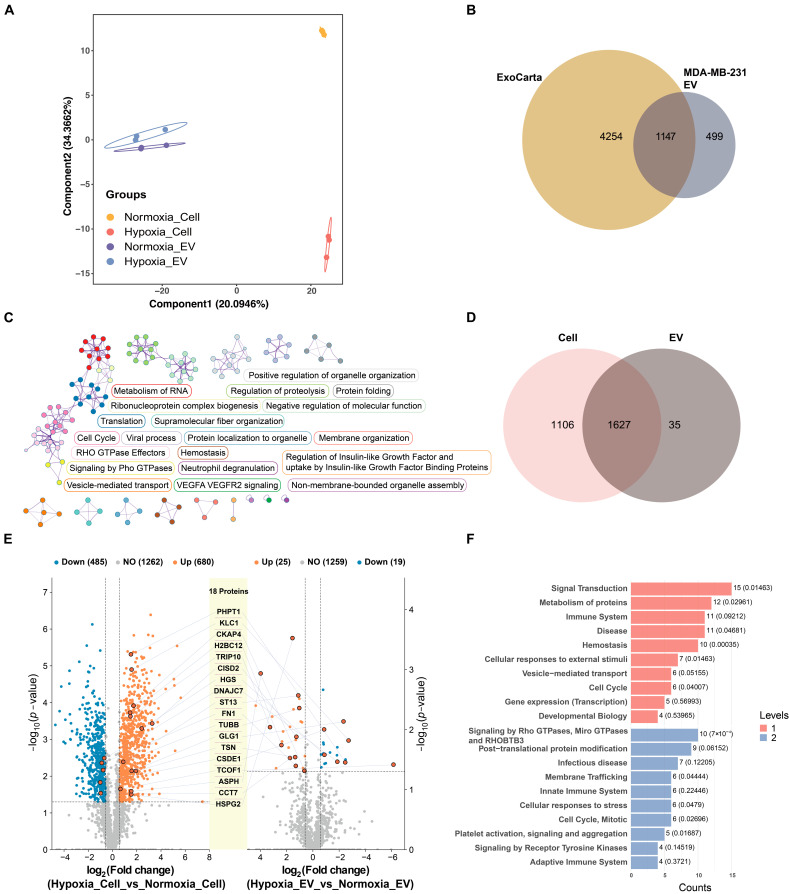
Proteomic analysis of EVs and cells under normoxic and hypoxic conditions. (**A**) Scatter plot of PLS-DA analysis of proteome profiles of normoxia_Cell (yellow circles), hypoxia_Cell (orange circles), and normoxia_EV and hypoxia_EV (purple and cyan squares). Symbols and ellipses indicate each sample and the 95% confidence interval of the four clusters. R^2^Y = 0.661; Q^2^Y = 0.574; *p* = 0.05. (**B**) Venn diagram showing comparison of protein groups identified in MDA-MB-231 cell EVs by LC–MS/MS with ExoCarta, a public EV proteomics database. Overlapping parts represent shared proteins, and non-overlapping parts represent unique proteins. (**C**) The network displays enriched biological pathways identified from EV proteome using Metascape. Nodes represent enriched terms, edges denote similarities, and colors indicate cluster IDs, highlighting different functional groups. (**D**) Venn diagram shows protein groups identified in cells and EVs using LC–MS/MS procedures (n = 6 per group). (**E**) Volcano plots showing differentially expressed proteins between normoxic and hypoxic conditions in both cells and EVs (*p* < 0.05 and fold change > 1.5; *q*-value < 0.05). The left panel presents a volcano plot of differentially expressed proteins between normoxic and hypoxic cells, while the right panel shows the differentially expressed proteins between normoxic and hypoxic EVs. The central diagram highlights the overlapping differentially expressed proteins identified in both conditions. (**F**) The top 20 entries of the Reactome pathway enriched for levels 1 and 2 by the differentially expressed proteins between normoxic and hypoxic EVs. Levels 1 and 2 indicate distinct biological themes enriched in the dataset.

To compare the proteomes of cells and EVs between hypoxic and normoxic conditions, the cells and EVs were digested in solution and analyzed by nano-LC–MS/MS. The proteomic analyses quantified 3392 proteins (Table S4) in the cells and EVs, which were then analyzed by PLS-DA. The PLS-DA showed a segregation of each group, with component 1 accounting for 20.09% and component 2 accounting for 34.37% of the variance (Figure 4A). In total, 1662 proteins were identified from all the isolated EVs. Among these, 1147 proteins (69.68%) overlapped with the EV proteome from the exosome database ExoCarta (Figure 4B). This result indicates that the procedures for isolation and purification are reproducible and that the results of the proteomics analysis are reliable. The proteomics analysis of the EVs using Metascape revealed enrichment in the critical biological pathways, including RNA metabolism, organelle organization, proteolysis regulation, and vesicle-mediated transport, alongside those pathways directly relevant to cancer progression, such as RHO GTPase signaling, VEGFA-VEGFR2 signaling, and cell cycle regulation (Figure 4C). A total of 2733 proteins were identified from the cell lysates. There were 35 protein groups unique to the EVs and 1627 protein groups common to both the EVs and cells (Figure 4D). Within the EVs under hypoxic conditions, 44 proteins exhibited higher abundance compared to those in normoxia (*p* < 0.05; fold change >1.5; *q*-value < 0.05) (Figure 4E; Table S5). Compared to the normoxic cells, 680 proteins were upregulated, and 485 proteins were downregulated in the hypoxic cells (*p* < 0.05; fold change > 1.5; *q*-value < 0.05) (Figure 4E). Notably, 18 dysregulated proteins were found to be overlapping between the cells and EVs under normoxic and hypoxic conditions. The Reactome pathway analysis of the altered proteins in the hypoxic versus normoxic cells revealed significant enrichment in the pathways related to protein metabolism, immune system responses, signal transduction, RNA metabolism, cellular stress responses, developmental biology, and vesicle-mediated transport (Figure S3). Furthermore, the Reactome pathway analysis of the dysregulated proteins in the hypoxic versus normoxic EVs highlighted notable involvement in the cell cycle, vesicle-mediated transport, and signaling by Rho GTPases, reflecting adaptational responses to hypoxic stress in the context of cancer pathophysiology (Figure 4F).

### 2.5. Correlation Analysis of N-Glycosylation-Related Proteins and Aberrant N-Glycan Profiles in Hypoxic Breast Cancer Cells and Their EVs

**Figure 5:**
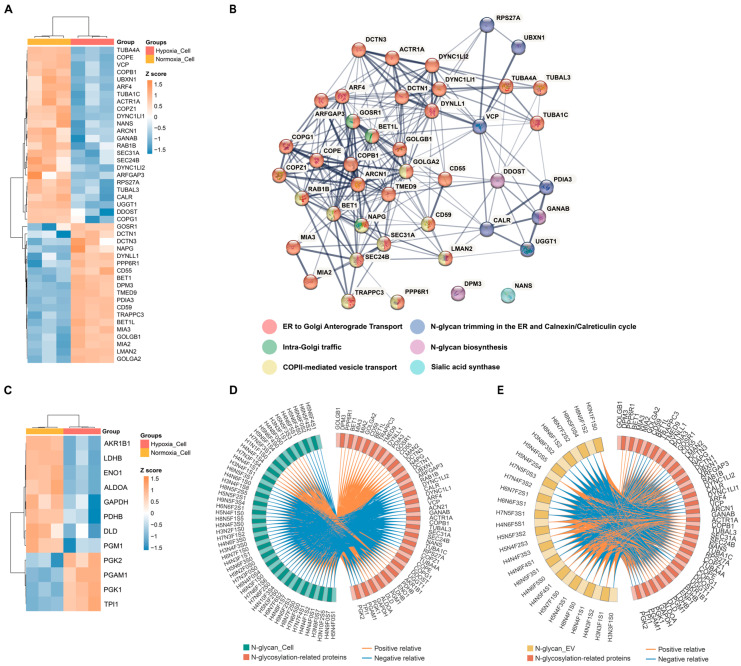
The expression profile and functional enrichment of glycosylation-related proteins in normoxic and hypoxic cells. (**A**) Heatmap of the differentially expressed “Asparagine N-linked glycosylation” proteins in hypoxic cell compared to normoxic cell. (**B**) STRING network analyses of significant differentially abundant proteins involved in asparagine N-linked glycosylation. (**C**) Heatmap of the differentially expressed glycolysis/gluconeogenesis proteins in hypoxic cell compared to normoxic cell. (**D**,**E**) Correlation Circos plot of differentially expressed N-glycans in EVs and cells under normoxic and hypoxic conditions and dysregulated N-glycosylation-related proteins in cells. Pearson coefficient cut-offs set at ≥0.8 or ≤−0.8. Blue lines represent negative correlations, while orange lines represent positive correlations.

Understanding the alterations in glycan-related-protein expression/abundance in response to hypoxia in breast cancer cells may enhance the exploration of the relationship between glycosylation and cancer progression. Integrating the ‘Asparagine N-linked glycosylation’ gene set from Gene set enrichment analysis (GSEA) identified 99 proteins associated with N-glycosylation in the cell proteome (Figure S4). Based on the differential expression analysis, 19 and 24 proteins were found to be significantly higher and lower in abundance in the hypoxic cells compared to the normoxic cells, respectively (all *p* < 0.05; fold change > 1.5; *q*-value < 0.05) (Figure 5A; Table S5). Reactome pathway enrichment analysis revealed these proteins’ involvement in endoplasmic reticulum (ER)-to-Golgi transport, N-glycan processing and modification, vesicle trafficking, and quality control within the asparagine-linked N-glycosylation pathway (Figure 5B).

Accompanying the protein-focused analyses, our research also delved into the metabolomic changes associated with altered glycosylation under hypoxia. Indeed, 12 dysregulated proteins were involved in the glycolysis/gluconeogenesis pathway by GSEA (all *p* < 0.05; fold change > 1.5; *q*-value < 0.05) (Figure 5C). We have identified seven key dysregulated metabolites from the published work by Yang, J. et al. [13], which are specifically relevant to the pathways of glycolysis and gluconeogenesis—processes known to intersect significantly with N-glycosylation dynamics. Integrating these metabolite alterations with the dynamics of the 12 dysregulated glycolytic proteins presents a nuanced view of the cellular metabolism in post-translational modifications during hypoxic stress (Figure S5). The correlation between the cellular proteins involved in the asparagine N-linked glycosylation and glycolysis/gluconeogenesis pathways with the aberrant N-glycans observed in the cells and EVs under hypoxic conditions was analyzed (Figure 5D,E; Table S6). The correlation heatmap revealed significant positive and negative correlations, indicating clear associations between these proteins and the N-glycan profiles of the EVs in response to hypoxia. Our findings suggest that the proteins involved in the asparagine N-linked glycosylation and glycolysis/gluconeogenesis pathways may play a pivotal role in modulating N-glycan profiles under hypoxic conditions.

## 3. Discussion

Glycosylation is a complex, highly regulated post-translational modification that is associated with both physiological and pathological conditions [14]. In highly proliferative solid tumors, low oxygen levels prompt cancer cell adaptation towards a more aggressive, chemoresistant, and radioresistant phenotype [2]. Hypoxia significantly influences glycosylation enzymes, resulting in altered glycan profiles [1]. Tumor-derived EVs enhance cancer cell adaptation to hypoxic conditions [4], but the effects of hypoxic stress on N-glycan dynamics and their loading into EVs to modulate cancer progression are not well understood. In this study, we isolated EVs from breast cancer cells via ultracentrifugation and analyzed the role of N-glycosylation in the hypoxia-induced EVs using proteomics and N-glycomics via nano-LC–MS/MS. We characterized the N-glycan profiles of the EVs and cells under both normoxic and hypoxic conditions. Our results indicate that tumor hypoxia induces dynamic changes in the N-glycosylation-related proteins and cellular N-glycan profiles, which may affect tumor malignancy and potentially impact patient outcomes. Oxygen deprivation triggers the synthesis of specific subsets of EV N-glycans that profoundly alter cancer cell migration and invasive potential. These insights into hypoxia-induced N-glycosylation alterations identify potential biomarkers and therapeutic targets for cancer treatment.

Since glycosylation is non-template-driven, it is influenced by various factors, including the expression efficiency of glycosyltransferase enzymes within the ER or Golgi, sugar nucleotide transporters, and the availability of sugar nucleotides [14]. Hypoxia affects the expression and functional activity of glycosyltransferases and the synthesis and transport of monosaccharides to the ER and Golgi, indirectly altering N-glycosylation [1]. Under hypoxic conditions, 43 asparagine N-linked glycosylation-related proteins in a cell influence various aspects of N-glycosylation, such as biosynthesis, ER trimming, and the calnexin/calreticulin cycle, as well as ER to Golgi transport and sialic acid synthesis. Hypoxia also stimulates glycolysis in cells, subsequently altering the availability of crucial substrates for N-glycan synthesis, thus impacting key cellular functions and interactions [1]. The dysregulation of the metabolic pathways is further exemplified by changes in critical metabolites like glucose, fructose, glucose-1-phosphate, and glucose-6-phosphate, which enhance glycolytic flux. Concurrent alterations in glycolysis/gluconeogenesis-related proteins in cells such as GAPDH, PGM1, and ALDOA influence substrate availability and nucleotide sugar synthesis, further affecting N-glycosylation and potentially leading to modifications in cellular behavior that are crucial for cancer progression.

Further understanding the effects of hypoxia on glycosylation and the resulting alterations in glycosylation-related proteins can provide critical insights into the N-glycan profiles produced by tumor cells, holding significant diagnostic and therapeutic potential. The analysis of the N-glycan patterns in breast cancer cells under hypoxic conditions demonstrates significant alterations closely tied to the mechanisms of cancer progression and metastasis. Utilizing LC–MS/MS, we distinguished 231 N-glycan patterns in the cells. N-glycan biosynthesis results in various glycosylation traits, such as branching, sialylation, and fucosylation, which critically influence cancer development by modulating cell signaling, adhesion, and the immune evasion mechanisms [5]. Notably, the relative abundance of high-mannose glycans, specifically Man3 (H3N2F0S0) and Man9 (H9N2F0S0), was elevated in the hypoxic cells. These N-glycans are frequently associated with breast cancer metastases, with Man9 linked to worse clinical outcomes in high-grade tumors [15,16] and Man3 showing increased levels in benign breast tumor tissues compared to para-carcinoma tissues [17]. Our results also highlight significant changes in the sialylation and fucosylation of N-glycans, emphasizing the importance of terminal modifications under hypoxia. For instance, the level of H3N5F1S0 was notably reduced in the hypoxic cells compared to in the normoxic conditions, a change implicated in the processes leading to breast cancer metastases to the brain [18]. Conversely, H5N4F0S1 was elevated in those cells under hypoxic conditions, exhibiting patterns similar to those observed in highly metastatic cells [18]. To investigate the underlying mechanisms, we analyzed the role of the proteins involved in the N-glycosylation-related pathways in cellular N-glycan alterations. These proteins showed high correlations with the dysregulated N-glycans in the hypoxic cells. Collectively, these findings illustrate that hypoxia significantly affects the expression of various N-glycan species by modulating the pathways involved in glycosylation biosynthesis. These N-glycosylation changes are potentially pivotal in facilitating brain metastasis and enhancing the overall metastatic progression in cancer.

EVs carry cargo that closely reflects a similar composition to their parent cells but can be modulated by microenvironmental factors such as hypoxia [19]. The weak correlation reflected by a low Pearson’s correlation coefficient between significantly differential abundant cellular and vesicular N-glycans under hypoxia suggests that, during EV formation, selective sorting mechanisms are at play, allowing specific glycosylation patterns to be preferentially packaged into EVs, leading to different N-glycan patterns in cells versus EVs. This selective sorting may serve to equip EVs with distinct biological functions tailored to the hypoxic tumor microenvironment, thus impacting processes like intercellular communication, migration, and invasion. Particularly in our result, approximately 85% of the N-glycans are overlapping in both the cells and EVs, while 27 unique N-glycan patterns were found only in the EVs, predominantly complex fucosylated and sialylated N-glycans. Specifically, N-glycan H7N6F1S2, absent in the MDA-MB-231 cells but present in the EVs, was linked to high diagnostic accuracy in breast cancer [20]. This highlights the potential of unique EV N-glycan profiles as biomarkers for cancer invasion and metastasis [18].

Notably, the relative abundance of mono-antennary N-glycans, specifically H4N3F1S2, H3N3F1S0, and H7N4F3S2, was significantly elevated in the hypoxic EVs, suggesting dysregulation or incomplete maturation of N-glycan biosynthesis associated with altered cell cycle dynamics, which reflects the heightened cellular proliferation in hypoxia [21]. Moreover, complex N-glycans with varied branching and fucose residues indicate intricate N-glycan synthesis pathways influenced by hypoxic stress. Specific N-glycan structures, like H6N4F1S1 and H5N4F1S1, showed significant alterations in the hypoxic EVs, suggesting their involvement in enhancing breast cancer invasiveness and potential as therapeutic targets [20,22]. Upregulated N-glycans like H8N4F1S0 and H8N6F1S2 in the hypoxic EVs suggest that these structures support cell–cell communication and promote survival under hypoxic conditions, which is potentially enhanced during cancer progression and metastasis [11]. Specifically, the significant expression of these N-glycans in both chemoresistant and chemosensitive cell lines under hypoxia underscores their potential involvement in cancer cells’ adaptive responses to hypoxic stress. Targeting the glycosylation pathways may disrupt the adaptive mechanisms of cancer cells in hypoxic tumor microenvironments [1]. Our correlation analysis between the differentially expressed N-glycans and dysregulated N-glycosylation-related proteins in the cells and EVs showed that the dysregulated N-glycans in the hypoxia-induced EVs display positive and negative correlations with the abundance of specific cellular proteins involved in N-glycosylation synthesis and glycolysis. For instance, the quantitative reduction in sialylation observed in the hypoxia-induced cells and EVs can be attributed to the downregulation of NANS expression, an enzyme crucial for N-acetylmannosamine-6-phosphate to N-acetylmannosamine-9-phosphate, a pivotal step in the biosynthesis of endogenous sialic acid [23]. Notably, DPM3 emerges as a central component of the dolichol phosphate–mannose (DPM) complex, which is essential for synthesizing DPM, a key precursor for the biosynthesis of high-mannose N-glycans within the endoplasmic reticulum [24]. Importantly, our findings indicate a strong correlation between DPM3 and the high-mannose N-glycan Man9, thereby emphasizing its crucial role in the glycosylation pathway. Although our analysis implicates correlations between the N-glycan profile and the abundance of proteins involved in the asparagine N-linked glycosylation and glycolysis/gluconeogenesis pathways, the molecular mechanisms must be investigated further.

## 4. Materials and Methods

### 4.1. Cell Lines and Cell Culture

The MDA-MB-231 breast cancer cell line was purchased from ATCC (Cell Lines Service, Eppelheim, Germany). MDA-MB-231 cells were cultured in DMEM supplemented with 10% FCS and 2 mM L-glutamine (Merck, Darmstadt, Germany). Cells were maintained at 37 °C in a humidified atmosphere with 5% CO_2_. Hypoxia treatment was performed at 37 °C in a water-saturated atmosphere at 5% CO_2_ in the incubator Heracell 15 (Thermo Fisher Scientific, Waltham, MA, USA) at 1% O_2_. The oxygen partial pressure was adjusted by N_2_.

### 4.2. EV Isolation

EV donor cells (MDA-MB-231) were slowly adapted to serum-free DMEM. Culture supernatants (100 mL) from cells cultured for 48 h under normoxia or hypoxia were collected, and protease inhibitors were added. The supernatants were centrifuged for 10 min at 500× *g*, followed by another 10 min at 5500× *g* to remove cells and cell debris. To isolate EVs, the supernatant was passed through a 0.22 µm and 0.1 µm sodium acetate filter (Cytiva, Freiburg, Germany). EVs were isolated by ultracentrifugation (70 min at 100,000× *g*, 4 °C) in an Optima L-100 XP Ultracentrifuge (Beckman Coulter, Brea, CA, USA) using an SW40Ti rotor following the procedure outlined by Santra et al. [25].

### 4.3. Nanoparticle Tracking Analysis (NTA)

Cell-culture-derived EVs were diluted with PBS 1:500 or 1:1000. Five videos of 30 s each were recorded (settings: camera level = 16, screen gain = 2) using the NanoSight microscope (LM14, Amesbury, UK). The NanoSight NTA 3.0 software’s processing function was used to analyze the recordings (settings: detection threshold = 6, screen gain = 2). PBS after 0.1 µm sodium acetate filter was also analyzed as background control and showed almost no signal.

### 4.4. Western Blot Analysis and Antibodies

MDA-MB-231 cells and their derived EVs were lysed at 4 °C using ice-cold sodium deoxycholate (SDC, (Merck, Darmstadt, Germany)) lysis buffer containing 100 mM triethylammonium bicarbonate and 1% (*w*/*v*) SDC. Protein extracts were analyzed via SDS-PAGE and Western blotting, with protein concentrations determined using the Pierce BCA Protein Assay Kit (Thermo Fisher Scientific, Dreieich, Germany). Protein lysates were mixed with NuPAGE LDS Sample Buffer (Thermo Fisher Scientific, Dreieich, Germany), heated for 10 min at 99 °C, and equal amount of protein was resolved in a NuPage 12% Bis Tris gel (Thermo Fisher Scientific, Dreieich, Germany). Following electrophoretic separation, proteins were transferred onto nitrocellulose membranes (Thermo Fisher Scientific, Dreieich, Germany) via wet blotting and blocked for 45 min with RotiBlock (Roth Chemie, Karlsruhe, Germany) in Tris-buffered saline with 0.1% Tween 20 (Merck, Darmstadt, Germany) containing 5% skimmed dry milk (Merck, Darmstadt, Germany) under gentle agitation at room temperature. The membranes were then incubated overnight at 4 °C with primary antibodies (diluted 1:1000), including anti-HIF1α (610958, BD Biosciences, Franklin Lakes, NJ, USA), anti-CD81 (56039T, Cell Signaling Technology, Danvers, MA, USA), anti-flotillin-1 (610820, BD Biosciences, Franklin Lakes, NJ, USA), anti-Lamin C (AB108595, Abcam, Cambridge, UK), and β-actin (4967S, Cell Signaling Technology, Danvers, MA, USA). After washing, the membranes were incubated with HRP-conjugated anti-mouse IgG (1:10,000) (LI-COR Biosciences, Lincoln, NE, USA) or anti-rabbit secondary antibody (1:3000) (LI-COR Biosciences, Lincoln, NE, USA). Protein bands were visualized using SuperSignal West Femto substrate (Thermo Fisher Scientific, Dreieich, Germany) with a ChemiDoc imaging station (BioRad, Feldkirchen, Germany), and densitometric quantification was performed using Quantity One software (Version 4.6.8, BioRad, Feldkirchen, Germany).

### 4.5. Mass Spectrometry-Based Proteomics and N-Glycomics

#### 4.5.1. N-Glycan Release and Peptide Preparation

Further, 50 µg of protein per cell lysate sample and 40 μg of protein per EV lysate sample were both diluted in SDC lysis buffer (0.1 M TEAB with 1% SDC) to a final volume of 100 µL. Subsequently, the samples were reduced and alkylated with 10 mM dithiothreitol (DTT, Sigma Aldrich, St. Louis, MO, USA) for 30 min at 56 °C and 20 mM iodoacetamide (IAA, Sigma Aldrich) for 30 min at 37 °C in the dark. Concentration and buffer exchange were performed using amicon ultra centrifugal filters filled with 100 mM ammonium bicarbonate. Protein digestion was performed by adding trypsin to each sample at a ratio of 1:100 and incubating at 37 °C for 18 h. N-glycans were cleaved from peptides using thirty units of PNGase F followed by enzymatic processing. The samples were vacuum-dried and then resuspended in 200 μL of 5% (*v*/*v*) acetic acid for the purification of N-glycans using the Sep-Pak C18 cartridge. Native N-glycan and peptide samples were dried using a SpeedVac concentrator (Thermo Fisher Scientific, Dreieich, Germany).

#### 4.5.2. N-Glycan Reduction and Solid-Phase Permethylation

N-glycan reduction and permethylation were performed following Guan et al. [26]. Released N-glycans were treated with a borane–ammonia complex at 60 °C for 1.5 h to remove α and β anomers from the reducing end. The reduced N-glycans were subsequently dried and re-dissolved in methanol to eliminate residual ammonium hydroxide and ammonium carbonate, drying using a SpeedVac concentrator. The permethylation of N-glycans was then performed using an optimized solid-phase method. Briefly, N-glycans were dissolved in DMSO/water solution, combined with methyl iodide, and incubated with NaOH beads at room temperature. The permethylation and any subsequent oxidation reactions were quenched with 5% acetic acid, and permethylated N-glycans were extracted using a chloroform–water separation method. The chloroform phases containing the permethylated N-glycans were vacuum-dried and stored at −40 °C for subsequent analysis or measurement.

#### 4.5.3. Peptide Purification by Single-Pot Solid-Phase-Enhanced Sample Preparation (SP3)

The dried peptides were purified using the SP3 protocol, as described by Hughes, C.S., et al. [27]. Briefly, the dried peptides were resuspended in 95% ACN, and 5 μL of SP3 beads (magnetic beads) were followed by vortexing and settling on the magnetic rack. The sample was washed twice with 500 μL of ACN, and the peptides were eluted with 50 μL of 2% DMSO/1% FA. The elution was centrifuged at 16,000× *g* for 5 min, and the supernatant was moved to a new tube. The samples were dried in a vacuum microcentrifuge concentrator.

#### 4.5.4. Analysis of N-Glycans and Peptides on Nano-LC–ESI-MS/MS

Analysis of reduced permethylated N-glycans and label-free peptides was performed using mass spectrometry under the same conditions as previously described by Godbole S et al. [28]. Briefly, chromatographic separation of peptides and N-glycans was achieved with a two-buffer system (buffer A: 0.1% FA in H_2_O; buffer B: 0.1% FA in ACN) on a nano-UHPLC (Dionex Ultimate 3000 UHPLC system, Thermo Fisher). Attached to the UHPLC was a peptide trap (100 µm × 20 mm, 100 Å pore size, 5 µm particle size, C18, Nano Viper, Thermo Fisher) for online desalting and purification, followed by a 25 cm C18 reversed-phase column (75 µm × 250 mm, 130 Å pore size, 1.7 µm particle size, peptide BEH C18, nanoEase, Waters). Sample measurement was performed in randomized order, and injection of 100 ng HeLa protein digest standard (88329, Thermo Fisher Scientific) was used for quality control before and after measurements.

Peptides were separated using an 80 min method with linearly increasing ACN concentration from 2% to 30% ACN over 60 min. MS/MS measurements were performed on a quadrupole-ion-trap-orbitrap MS (Orbitrap Fusion, Thermo Fisher). Eluting peptides were ionized using a nano-electrospray ionization source (nano-ESI) with a spray voltage of 1800 V and analyzed in data-dependent acquisition (DDA) mode. For each MS1 scan, ions were accumulated for a maximum of 120 milliseconds or until a charge density of 2 × 10^5^ ions (AGC Target) was reached. Fourier-transformation-based mass analysis of the data from the orbitrap mass analyzer was performed covering a mass range of *m*/*z* 400–1300 with a resolution of 120,000 at *m*/*z* = 200. Peptides with charge states between 2+ and 5+ above an intensity threshold of 1000 were isolated within a *m*/*z* 1.6 isolation window in Top Speed mode for 3 s from each precursor scan and fragmented with a normalized collision energy of 30% using higher-energy collisional dissociation (HCD). MS2 scanning was performed using an ion trap mass analyzer at a rapid scan rate, covering a mass range starting at *m*/*z* 120 and accumulated for 60 ms or to an AGC target of 1 × 10^5^. Already fragmented peptides were excluded for 30 s.

For permethylated N-glycan analysis, a 115 min gradient was utilized, beginning with 2% solvent B, which increased to 30% over 10 min, followed by an increase to 75% over 70 min, and concluding at 95% by the end of the gradient. N-glycans were analyzed using a quadrupole–orbitrap–ion trap mass spectrometer in DDA mode. For MS1 scanning, an orbitrap mass analyzer with a resolution of 120,000 FWHM was used, with an AGC target of 2.0 × 10^6^ and an *m*/*z* scan range from 400 to 2000. For collision-induced dissociation (CID)-MS/MS, the most intense precursor ions were selected for fragmentation, isolated using a 2 m/z window, with a normalized collision energy of 35%. Fragments were detected at an orbitrap resolution of 17,500 FWHM, with an AGC target of 1.0 × 10^4^ and a maximum accumulation time of 20.00 ms. MS data are available via ProteomeXchange [29] with identifier PXD053696 and GlycoPOST [30] with identifier GPST000466.

### 4.6. Processing of Proteome and N-Glycome Raw Data

Proteome raw data were searched with the Sequest algorithm integrated into the Proteome Discoverer (Version 3.0.0.757, Thermo Fisher Scientific) against a reviewed human Swissprot FASTA database, obtained in April 2021, containing 20,365 entries. Subsequent data processing and proteome analysis were conducted using Perseus (version 2.0.11.0).

Raw N-glycan data visualization was carried out with Xcalibur Qual Browser, and molecular masses were extracted utilizing MaxQuant. Potential monosaccharide compositions of N-glycan precursors were determined through an in-house Python script [26]. The identification of N-glycan compositions and structures was based on MS/MS results, with manual assignments correlating observed peaks in MS/MS spectra to potential N-glycan fragments listed in GlycoWorkbench 2.1. N-glycan compositions were annotated with specific symbols representing monosaccharide components (H (Hexose)-N (HexNAc + red-HexNAc)-F (Fucose)-S (Neu5Ac)). The determined N-glycan structures adhered to the Symbol Nomenclature for Glycans guidelines and were visually represented using GlycoWorkbench [31,32]. Quantitative analysis of N-glycans was facilitated using Skyline software 21.1.0.278, with peak area data subsequently exported for further analysis in Excel.

### 4.7. Statistical Analysis and Visualization

Relative protein abundances were log2-transformed and normalized to the median protein abundance in each sample using Perseus to correct for variations in sample-to-sample injected amounts. Protein-enriched pathways were assessed by the Genomes (KEGG) database and Reactome Knowledgebase [33] and the clusterProfiler R package 4.12.6 [34], with significant pathways identified at a *p*-value < 0.05. Identified EV proteins were compared with data from the ExoCarta database, and proteomic profiles of EVs were analyzed using Metascape for pathway enrichment [35]. GSEA was employed to identify and enrich protein sets related to “Asparagine N-linked glycosylation” and “Glycolysis/Gluconeogenesis” from cell proteome profiles. The functional interaction among significant expressed Asparagine N-linked glycosylation proteins was derived from the STRING database [36]. Additionally, MetScape was employed for the integration and visualization of dysregulated metabolites alongside dysregulated proteins in the glycolysis/gluconeogenesis pathways to map interactions and biological pathways impacted in hypoxia-induced cells. Relative quantification of individual N-glycan species was performed by calculating their percentages relative to the total N-glycans detected, with traits such as truncation, high-mannose, hybrid-type, complex-type, number of antennas, bisecting GlcNAc, sialylation, fucosylation, galactosylation, poly-LacNAc motifs, Lewis A/X, and sLewis A/X analyzed [37]. Statistical assessments of significant changes in glycosylation features were performed using one-way ANOVA, and dysregulations in proteins and N-glycans were identified using the Student’s *t*-test with the Benjamini–Hochberg FDR adjustment method (*p*-value < 0.05; fold change > 1.5; *q*-value < 0.05). The nine-quadrant map analysis and Circos plot were both utilized to identify significant correlations between differentially expressed N-glycans in hypoxic vs. normoxic cells and hypoxic vs. normoxic EVs, and dysregulated N-glycosylation-related proteins using Pearson’s correlation coefficients. PLS-DA was employed to differentiate sample cohorts based on proteins and N-glycans. Data visualization, including volcano plots, heatmaps, Venn diagrams, and scatter plots, was performed using R 4.2.2 and GraphPad Prism software (Version 7.0), with results considered statistically significant at *p*-value < 0.05.

## 5. Conclusions

This study provides a detailed characterization of the N-glycosylation profiles of cells and EVs derived from MDA-MB-231 breast cancer cells under hypoxic conditions using nano-LC–MS/MS. We identified distinct N-glycan types, including mono-antennary structures and specific N-glycans such as H4N3F1S2, H3N3F1S0, H7N4F3S2, H8N4F1S0, and H8N6F1S2, which were enriched in the hypoxia-induced EVs. Additionally, high mannose glycans like Man3 and Man9 were prevalent in the hypoxic cells. These modifications suggest that N-glycosylation patterns unique to hypoxic conditions significantly influence tumor biology, potentially altering the interactions within the tumor microenvironment to promote tumorigenesis and metastasis. Our findings revealed the critical impact of hypoxia on modulating N-glycosylation-related proteins and altering glycan profiles, highlighting the adaptive mechanisms that support cancer cell survival and invasion. Understanding these N-glycosylation differences provides novel EV-based strategies to target breast cancer, such as exploiting the unique N-glycan signatures for selective isolation and targeting of tumor-derived EVs, enhancing immunotherapeutic approaches, and providing biomarkers for tumor hypoxia. This study elucidates the importance of hypoxia-induced N-glycosylation in breast cancer, offering a new tool for understanding and exploiting the tumor microenvironment in breast cancer management.

## Data Availability

The mass spectrometry proteomics data have been deposited to the ProteomeXchange Consortium via the PRIDE partner repository with the dataset identifier PXD053696. The mass spectrometry glycomics data have been deposited to GlycoPost repository with the dataset identifier GPST000466.

## Supplementary Materials

The supporting information can be downloaded at https://www.mdpi.com/article/10.3390/ijms251810216/s1.
