# Supplementary material for: Hypoxia-Induced Adaptations of N-Glycomes and Proteomes in Breast Cancer Cells and Their Secreted Extracellular Vesicles
Source: Int J Mol Sci. 2024 Sep 23;25(18):10216. doi: 10.3390/ijms251810216 (PMC11432262; doi:10.3390/ijms251810216)
Supplement: Supplementary file 1 [file ijms-25-10216-s001.zip › Supplementary_Figures_after-proofread.pdf]

Supplementary information

Contents

Supplementary Figure S1 2

Supplementary Figure S2 3

Supplementary Figure S3 4

Supplementary Figure S4 5

Supplementary Figure S5 6

Supplementary Figure S1

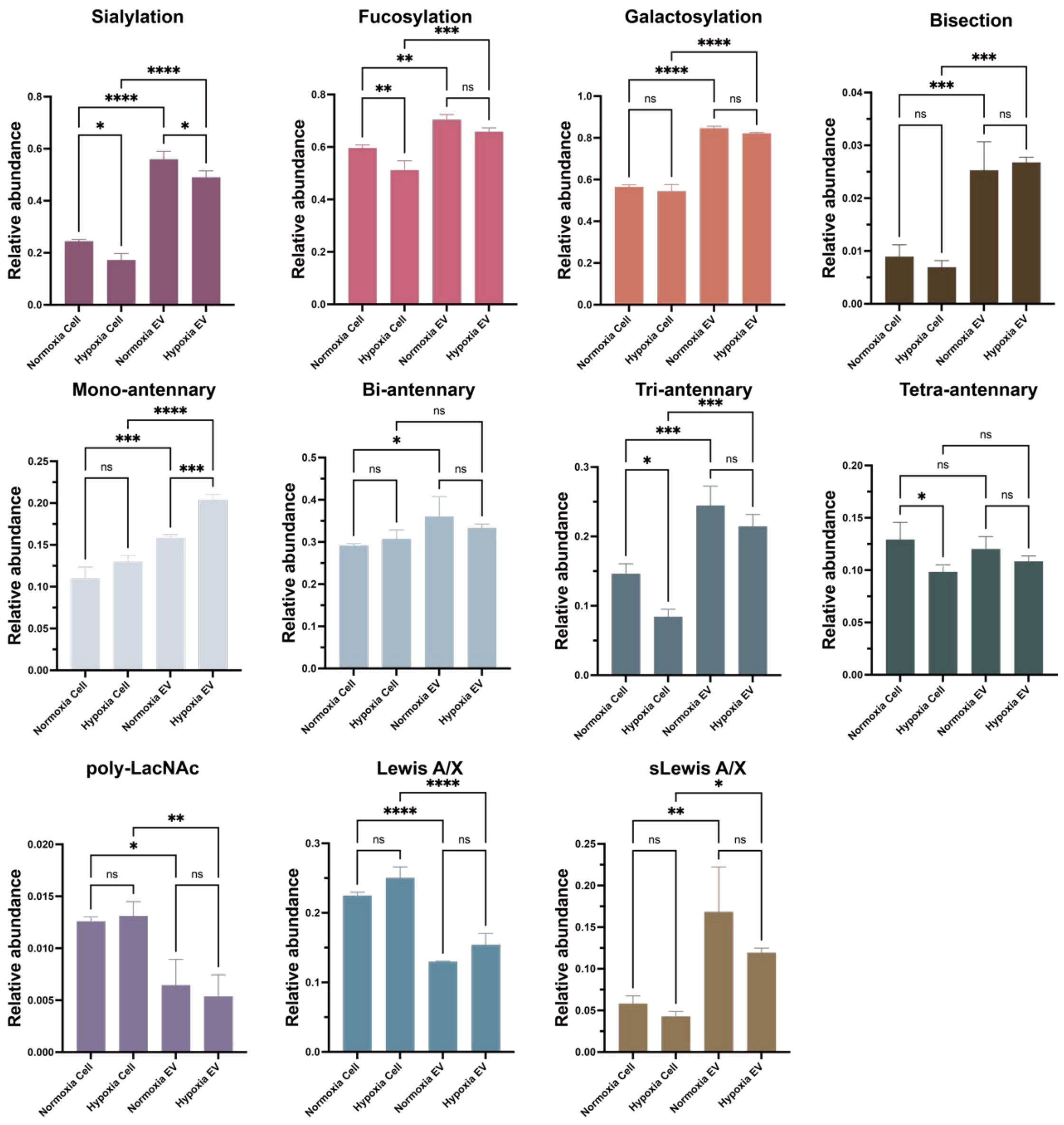

Figure S1 Distribution of derived N-glycosylation features in cell and EV under normoxic and hypoxic condition. Significant values are marked with ns (no significant), * ($p \leq 0.05$), ** ($p \leq 0.01$), *** ($p \leq 0.001$), and **** ($p \leq 0.0001$).

Supplementary Figure S2

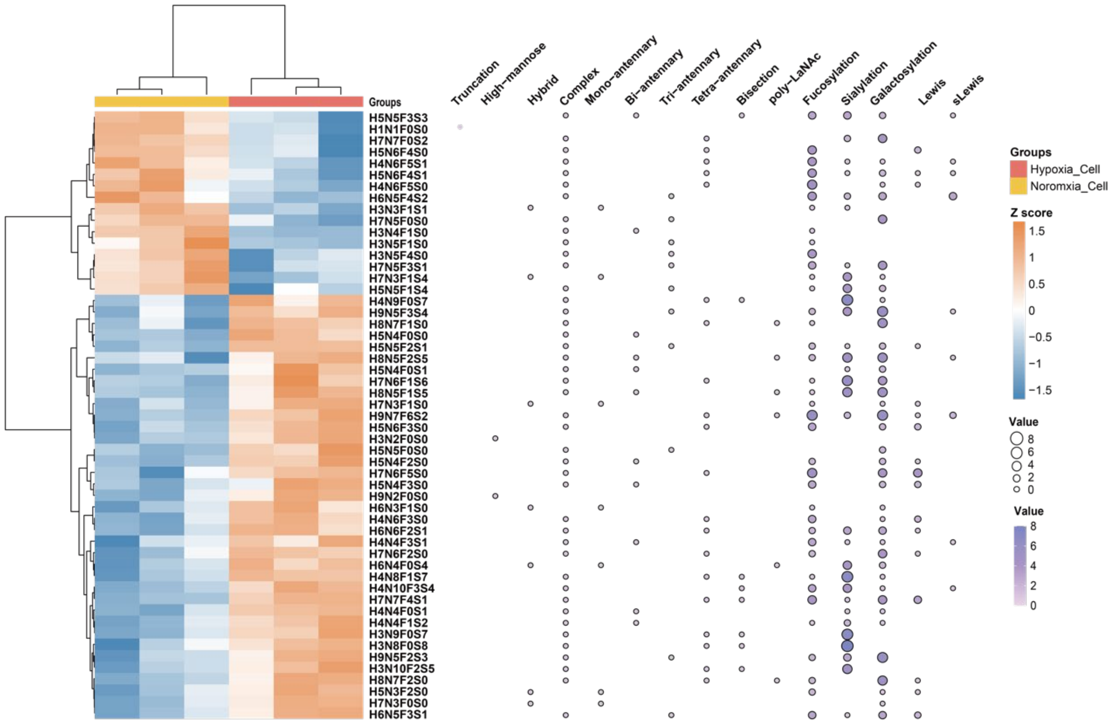

Figure S2 The heat map for the hierarchical clustering of all t-test significant N-glycans with different glycosylation traits in hypoxic *vs.* normoxic cells ($p < 0.05$, fold change > 1.5, and q-value < 0.05). 53 altered N-glycans were classified by biosynthetic class. Each N-glycan is assigned Ballon plot depicting glycosylation features in the respective N-glycan.

Supplementary Figure S3

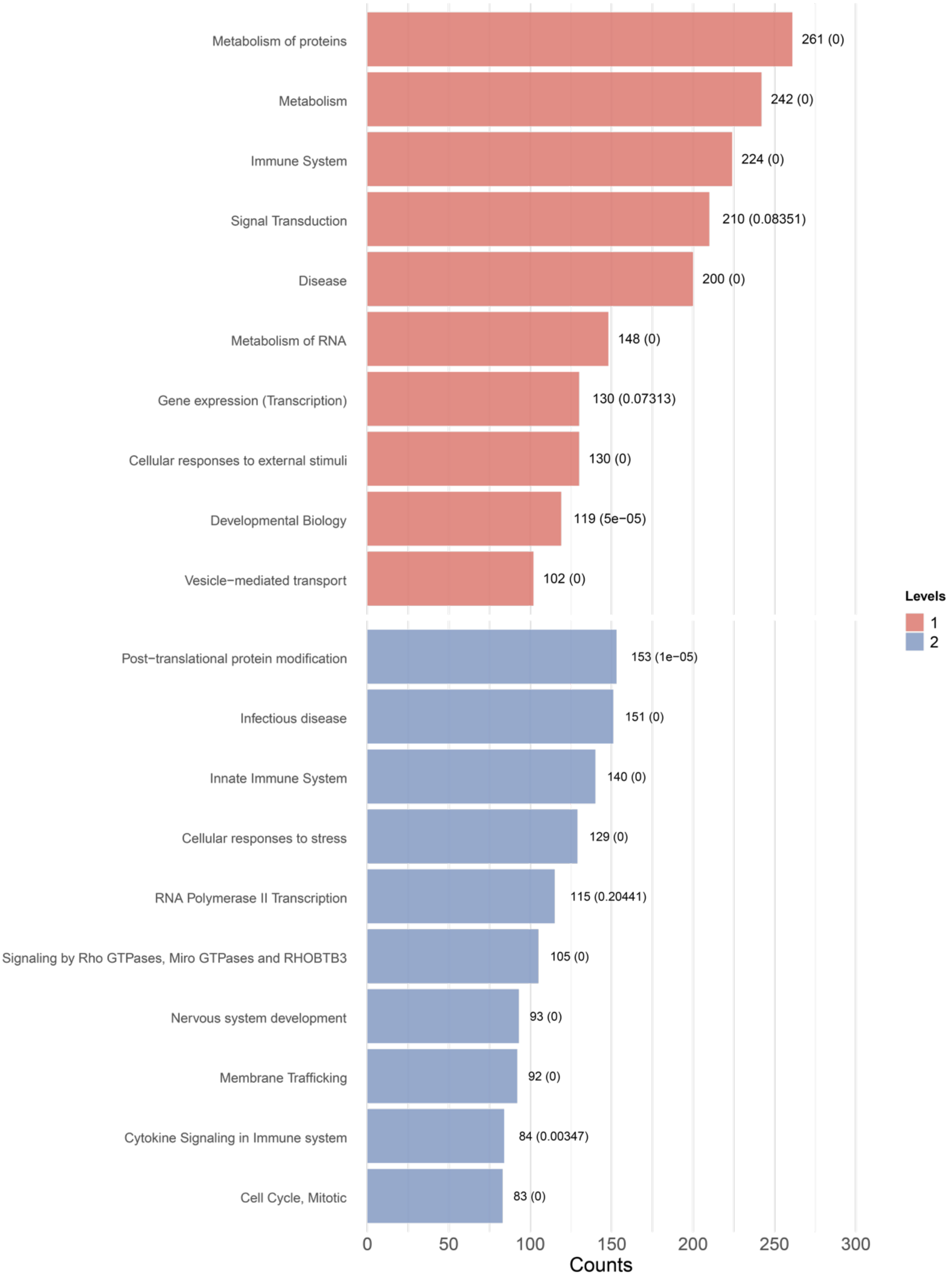

Figure S3 The top 20 entries of the Reactome pathway enriched for level 1 and 2 by the differentially expressed proteins between normoxic and hypoxic cells. Levels 1 and 2 indicate distinct biological themes enriched in the dataset.

Supplementary Figure S4

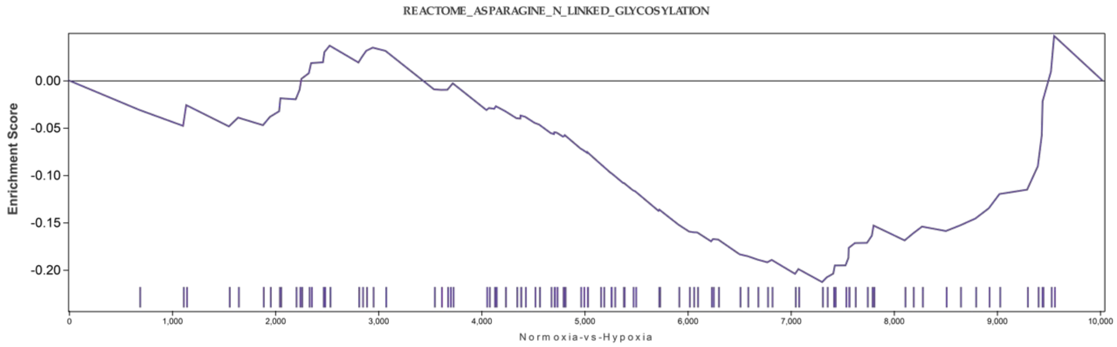

Figure S4 Genes set enrichment plots for Asparagine N-linked glycosylation in normoxic and hypoxic cells.

Supplementary Figure S5

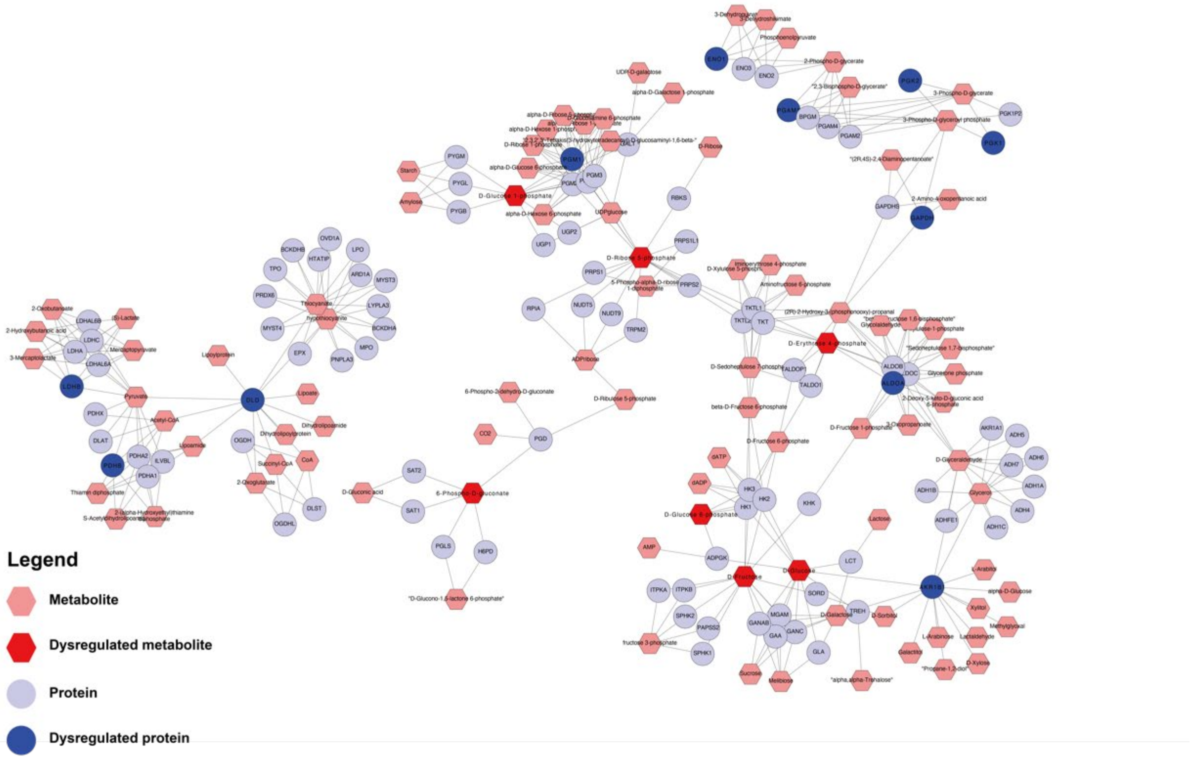

Figure S5 Network of the glycolysis/gluconeogenesis pathway from Metscape analysis corresponds to seven metabolites and 12 dysregulated glycolysis/gluconeogenesis proteins.
